# Peripartum Cardiomyopathy: Moving Towards a More Central Role of Genetics#

**DOI:** 10.2174/1573403X113099990029

**Published:** 2013-08

**Authors:** Roberto Cemin, Rajesh Janardhanan, Luca Donazzan, Massimo Daves

**Affiliations:** a Department of Cardiology, San Maurizio Regional Hospital, Bolzano, Italy; b Clinical Biochemical Laboratory, San Maurizio Regional Hospital, Bolzano, Italy; c Department of Cardiology, University of Arizona, Tucson, AZ, USA

**Keywords:** Peripartum cardiomyopathy, heart failure, echocardiogaraphy.

## Abstract

Peripartum cardiomyopathy (PCM) is a relatively rare disease with potentially devasting consequences requiring prompt identification and correct treatment. Overall prognosis is good in majority of the cases, although some patients may progress to irreversible heart failure. Early diagnosis is important and effective treatment reduces mortality rates and increases the chance of complete recovery of ventricular systolic function.

The aetiology and pathogenesis seems to be multifactorial and poorly understood, with the available literature rather conflicting. In recent years, there has been increased interest in the role played by genetic predisposition in the development of PCM. It probably develops as a result of a complex interaction of pregnancy-associated factors and genetic factors and recently there have been many observations pointing out the central role played by a genetic predisposition. The direct and indirect observations on genetic susceptibility may offer new insights into the pathogenesis of PCM. However, larger studies are needed before advising routine genetic testing in these patients.

## INTRODUCTION

Although diagnostic criteria for peripartum cardiomyopathy (PCM) were established many decades ago [2] its aetiology remains unclear. The principal hypotheses with regards to the pathogenesis of this cardiomyopathy include an autoimmune response, an abnormal reaction to physiologic hormones, an abnormal response to haemodynamic stress of pregnancy or a viral aetiology [3-6]. Recently, increasing evidence about a central role played by genetics has been shown [7, 8]. PCM is a syndrome with symptoms of heart failure and signs of left ventricular systolic dysfunction, which manifest between the last month of pregnancy and the first 5 months postpartum [9]. Rarely it could present earlier during pregnancy and therefore the strict temporal limitation in the definition has been recently removed [10] and changed to “towards the end of pregnancy or in the months following the delivery”. 

Its incidence varies from 0.2‰ to 3‰ live births [9, 11, 12] and from region to region worldwide. The prognosis is generally good in the majority of the cases although some patients progress to irreversible heart failure, heart transplantation or death [12, 13]. The diagnosis of PCM is made in the presence of symptoms and signs of heart failure strictly associated with partum and in the absence of other possible causes of dilated cardiomyopathy. Presence of ventricular systolic dysfunction is essential for the diagnosis. Some echocardiographic parameters like the presence of an ejection fraction of less than 45% and an end-diastolic dimension index of greater than 2.7 cm/m^2^ have been proposed to better classify the dysfunction [14]. 

## CLINICAL FEATURES

PCM usually presents with classical symptoms and signs of systolic heart failure with ventricular enlargement and dysfunction demonstrated by echocardiography. Often there is associated significant mitral and tricuspid regurgitation [15]. Unusual presentations include thromboembolism or hepatic failure. In more than 90% of the cases, the time of diagnosis and development of heart failure is usually post-partum [16]. PCM can occur at any age with a higher incidence in women older than 30 years [2, 13]. 

## PATHOGENESIS

The aetiology and pathogenesis seems to be multifactorial and poorly understood with the available literature rather conflicting. There is probably a complex interaction between certain risk factors, abnormal hormonal and immune response to pregnancy, abnormal response to hemodynamic stress of pregnancy with a genetic predisposition. The role played by a possible previous episode of myocarditis is still a matter of great debate. 

### Risk Factors

Gestational hypertension, tocolytic therapy and twin pregnancy have been proposed as possible risk factors because they were commonly associated with PCM [13]. However, some studies [17] showed no association between history of hypertension during pregnancy, use of tocolytc agents and PCM. Multiparity could be a potential risk factor for the development of PCM [18] but interestingly, some studies have shown that more than 50% of the patients are at their first or second pregnancy [13]. 

### Myocarditis

Some studies have proposed a recent episode of myocarditis as the possible trigger for PCM. Histological findings of myocardial biopsies could support this hypothesis showing evidence of previous myocarditis in 9-62% of the patients [19-23]. Although these findings could suggest an association between inflammation and PCM, no causal relationship has yet been determined.

### Autoimmunity

The association between PCM and twin pregnancy could support the theory of autoimmunity as a possible mechanism. This could depend on an excessive traffic of hematopoietic lineage cells from the fetus to the mother as manifest in twin pregnancy [24]. Usually the lower concentrations of these foreign proteins could contribute to tolerance of the fetus while increased levels could theoretically lead to the initiation of autoimmune disease [15]. The weak immunogenicity of the paternal haplotype of the chimeric cells or the naturally immunosuppressive state of the mother or both could avoid rejection of fetal cells during pregnancy. Postpartum, the recovery of immune competence could trigger a pathologic autoimmune response against cardiac cells where hematopoietic cells have taken up residence during pregnancy and therefore myocardial cells are recognised as nonself [9]. 

Additional evidence for an immune-mediated component of PCM is the production of plasma anti-cardiac antibodies in response to specific cardiac antigens [3]. However, characterization of the IgG subclass of anti-myosin heavy chain antibodies from PCM patients is less supportive of an auto-immune mediated process and more suggestive of an immune system dysfunction [25]. 

Whether autoimmunity actually plays a role in the pathophysiology of PCM or whether it is a consequence of cardiac damage due to another mechanism remains uncertain [26]. 

### Inflammation

Molecular markers of an inflammatory process are found in most patients. 90% of the patients show high levels of plasma C-reactive protein that correlated positively with LV end-diastolic and end-systolic dimensions and inversely with LV ejection fraction [17]. This could indicate the chronic inflammatory state at baseline, which is more pronounced in unstable patients. The presence of a low-grade chronic inflammatory process could be due to the release of endotoxins and subsequent release of pro-inflammatory cytokines [27] or a consequence of an immune system dysfunction [25]. 

### Hormonal Abnormalities (Excessive Prolactin Production/Cleavage)

Women with acute PCM have increased serum concentrations of oxidised low density lipoprotein, which is a marker of oxidative stress. Recent data suggest that oxidative stress, prolactin and the prolactin-cleaving protease cathepsin D, may be implicated in the pathogenesis of PCM. Increased oxidative stress could lead to increased expression and proteolytic activity of cardiac cathepsin D. The last one cleaves prolactin into a potent antiangiogenic, proapoptotic and proinfiammatory factor (the 16kDa). The 16 kDa fragment inhibits endothelial cell proliferation and migration, induces endothelial cell apoptosis, disrupts capillary structures, promotes vasoconstriction and impairs cardiomyocyte function [17, 28]. 

## GENETICS

In recent years there have bee many indirect demonstrations of the role played by genetic predisposition in the development of PCM. Even the fact that the disease is more common in some regions of the world (i.e. South Africa 1:1000, Haiti 1:300 and Nigeria 1:100 vs 1:2000-4000 in the United States) [13, 15] should suggest a genetic implication in the aetiology of the disease. Environmental risk factors could also explain these differences, but genetics most likely plays an important role because the incidence remains higher in the Africans who moved to the United States. The incidence has been observed as 1 in 1421 among African Americans in California vs 1 in 9861 among Hispanics in California [12]. There is a 16-fold higher incidence noted in African American living in Georgia and Tennessee compared with non-African American women [29]. 

Another observation supporting the genetic role is the familial clustering of the disease [30, 31]. Cases of PCM observed in families at higher risk of idiopathic cardiomyopathy point to yet another clue of genetic involvement in the aetiology of PCM. A hereditary predispositon is also suggested by familial reports of PCM [32] and strong consideration should be given to screening family members because PCM may be the forme fruste of a genetic predisposition to cardiomyopathy [9]. 

It has been suggested that PCM could be part of a spectrum of familial dilated cardiomyopathies, unmasked earlier in life by the haemodynamic stress of pregnancy [33, 34]. A mutation in the gene encoding cardiac troponin C was identified in one family presenting with multiple cases of PCM and dilated cardiomypathy [34]. 

The first direct demonstration of an involvement of the genes has been obtained in mice [28]. A restricted deletion in the STAT-3 gene of cardiomyocyte in mice resulted in the development of PCM. A reduction in STAT-3 leads to increased oxidative stress and activation of cathepsin D, resulting in a detrimental effect on myocardial microvasculature and causing myocardial hypoxemia, apoptosis and development of PCM. 

The recent identification of a genetic mutation associated with this potentially fatal cardiomyopathy was an important step forward in understanding the mechanism underlying PCM. Women with PCM have been shown to be about two-and-a-half times more likely than healthy women to carry the genetic mutation of the chromosome 12p11.22 locus (nucleotide polimorphism of rs258415) [8]. That gene has been shown to be involved in regulating blood pressure, muscle contraction of the heart and in the uterus. Furtherome it is located near the PTHLH gene, which is involved in the lactation process and which is itself regulated by prolactin production. All these direct and indirect observations are really intriguing and provide insights into potential genetic aetiology of PCM

## PROGNOSIS

Overall prognosis of PCM is good in majority of the cases, although some patients may progress to irreversible heart failure. Progression of the condition requiring heart transplantation is described in 4% and death in 9% at a two years follow up [13]. Other studies showed a much higher mortality rate such as 15% or 32% at 6 months [35]. Patients who eventually die tend to have worser NYHA functional class, LVEF and larger LV dimensions at diagnosis [17]. There seems to be an initial high-risk period with 25-50% of the women dying within the first 3 months postpartum [36]. Sudden cardiac death has been reported to account for up to 50% of the mortality [22] and therefore attention should be paid to identify those patients who are likely to experience a late recovery of systolic function from those who should be considered for implantation of a cardioverter-defibrillator. Mortality rates have decreased over the past 10 years due to advances in medical therapy for heart failure and use of implantable defibrillators [37]. Normalisation of left ventricular systolic function occurs in 23% of the patients at six months [17] and 54% at two years in patients especially if EF at diagnosis is more than 30% [13]. Higher left ventricular end diastolic dimension and lower fractional shortening at diagnosis seem to be associated to a worse prognosis. A fractional shortening of less than 20% and a left ventricular end diastolic dimension of 6 cm or greater was associated with a more than 3-fold higher risk for persistent left ventricular dysfunction [38]. 75% of the patients who recover, have an EF of more than 45% at two months after diagnosis [39]. 

Since left ventricular recovery occurs in most patients in less than 6 months and because sudden cardiac death caused by ventricular arrhythmias is not rare in these patients, in some high risk patients it seems reasonable to consider temporary use of wearable external defibrillators as a bridge to recovery or ICD implantation. A final decision about permanent ICD implantation should be taken at 6 months, allowing the left ventricle to recover during medical therapy. 

Complete recovery of systolic function occurs usually in the first 6 months after delivery [12] although the recovery phase may not be limited to the first 12 months. Continuing improvement has been observed in the second and third year after diagnosis [11]. Persistence of the disease after 6 months portends worse prognosis and worse survival [40]. 

To determine prognosis at the time of diagnosis, a dobutamine stress echocardiography study could be performed in non-critically ill patients. Inotropic contractile reserve seems to accurately correlate with subsequent recovery of left ventricular function and is associated with a benign prognosis [36]. Even if left ventricular function recovers completely, exercise tolerance may remain abnormal and this could be more objectively assessed by an abnormal response to dobutamine stress echocardiography. 

There has been rare occurences of spontaneous late deterioration of left ventricular function after complete recovery. Hence annual echocardiographic examinations are suggested in all the patients.

## THERAPY

Management reflects conventional therapy for heart failure with diuretics, ACE-inhibitors, beta-blockers and angiotensin-receptor blockers. Anticoagulant therapy should be considered in view of the low left ventricular ejection fraction, which predisposes to thrombus formation, especially in the peripartum period when a hypercoagulable state exists. In patients not improving on conventional therapy or in patients with critical hemodynamic state with cardiogenic shock, hemodynamic support with pressors should be considered. There have been some reports about the use of levosimendan [41] in non-responsive patients.

Non-responders should be considered for heart transplantation even if there are some reports about effective extracorporeal membrane oxygenation [42], intraaortic balloon pump and use of mechanical assist devices. Since the rate of recovery in PCM patients is high, an attempt should be made to use these devices as a bridge to recovery before referring them to cardiac transplantation.

Among patients who did recover, the withdrawal of heart failure medications was not associated with decompensation over a follow-up of 29 months [39]. Patients with normal EF at rest and during dobutamine could taper off medical therapy in 6-12 months, while patients with abnormal EF during dobutamine should be treated for longer period with ACE-inhibitors and betablockers [43]. Patients who continue to have a depressed ventricular function have a poorer prognosis and should receive medical therapy indefinitely. In any case, it seems reasonable to continue at least for a year with ACE-inibitors and betablockers even if there is complete recovery.

It is important to note that the use of ACE-inibithors should be restricted to the post delivery since they have teratogenic effects. Other drugs like immunosoppressive drugs or pentoxifilline are still under evaluation.

Recently, a favorable response to bromocriptine, a pharmacological inhibitor of prolactin has been described in a limited number of patients with PCM. The use of bromocriptine is based on the observation of an enhanced oxidative stress-mediated cleavage of the nursing hormone prolactin into the antiangiogenic and proapoptotic 16-kDa fragment (see above), which could take part in PCM development. In case series involving very small number of patients, bromocriptine was shown to be useful in addition to standard heart failure therapy, promoting a significant larger rate of left ventricular function recovery at 6 months and a lower rate of mortality [31, 44-47]. Bromocriptine is associated with suppression of breast milk production and also increases the risk of thromboembolic phenomena, causing potential complication to the mother. Increased incidence of myocardial infarctions has been associated with bromocriptine use [48]. Hence at present, it is not recommended as standard treatment in patients with PCM. Larger randomized trials are needed, before this treatment can be recommended as a routine strategy. 

## SUBSEQUENT PREGNANCIES

A subsequent pregnancy carries a high risk of relapse, significant decrease of left ventricular function and mortality. Mortality rate is described to be 55.5% during subsequent pregnancy [49] even if it seems to be associated more with patients who entered the subsequent pregnancy with abnormal systolic function *i.e*. without making a complete recovery [50]. Complete recovery from a relapse is very rare. There is no consensus regarding recommendations for future pregnancy after PCM but patients whose left ventricular size or function does not return to normal should be counseled strongly to avoid subsequent pregnancy [9]. 

## CONCLUSION

PCM is a relatively rare disease which can have devasting consequences and should be promptly identified and correctly treated. Early diagnosis is important and therefore women who develop symptoms of heart failure in their pregnancy or shortly after should be investigated for this condition. Effective treatment reduces mortality rates and increases the chance of complete recovery of ventricular systolic function. PCM probably develops as a result of a complex interaction of pregnancy-associated factors and genetic factors (genetic susceptibility) and recently there have been many observations pointing to the central role played by a genetic predisposition. 

**Fig. (1):**
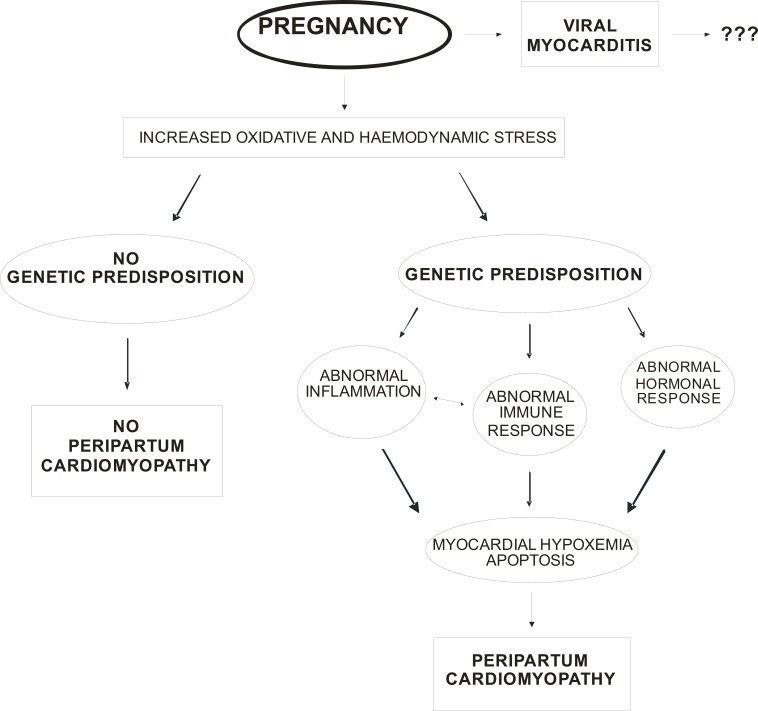
Proposal of unifying hypothesis for the pathogenesis of PCM: moving towards a more central role of genetics (see text for detail).

Increased oxidative and hemodynamic stress related to pregnancy, could bring to an abnormal immune and hormonal response in genetically predisposed women, causing abnormal myocardial inflammation, hypoxemia, apoptosis and, finally, the development of overt PCM (see Fig. **1**). The possible role of a previous myocarditis, even if proposed in the past, seems very doubtful because no causal relationship has yet been determined between previous myocarditis and PCM development. 

Genetic testing, although rapidly emerging into clinical practice, is currently undertaken at only a few centers [51]. This approach may be rapidly changing as and when more cost-effective screening methods become available. Time and cost, however, remain pertinent considerations [52] and routine genetic testing of PCM patients is not indicated at the moment. It could become potentially useful, especially in women considered at high risk who have relatives with PCM or dilated cardiomyopathies. Further large scale studies are necessary to confirm a central genetic role in the pathogenesis of PCM, which could lead to reclassification of this cardiomyopathy, with the possibility of including it into a genetic determined form, associated with pregnancy.
